# Factors influencing quality of life in patients followed in the neurosonology laboratory for carotid stenosis

**DOI:** 10.1186/s12955-018-0902-2

**Published:** 2018-04-27

**Authors:** Táňa Fadrná, Zdeňka Mikšová, Roman Herzig, Kateřina Langová, Libor Ličman, David Školoudík

**Affiliations:** 1 Department of Nursing, Faculty of Health Sciences, Palacký University Olomouc, Olomouc, Czech Republic; 2 Center for Science and Research, Department of Nursing, Faculty of Health Sciences, Palacký University Olomouc, Hněvotínská 3, CZ-775 15 Olomouc, Czech Republic; 3 Department of Neurology, Comprehensive Stroke Center, Charles University Faculty of Medicine and University Hospital Hradec Králové, Hradec Králové, Czech Republic; 4 Department of Biophysics, Faculty of Medicine and Dentistry, Institute of Molecular and Translational Medicine, Palacký University, Olomouc, Czech Republic

**Keywords:** Quality of life, Questionnaire, Stroke, Risk factors

## Abstract

**Background:**

Quality of life (QoL) is one of the main endpoints in stroke prevention or acute stroke treatment studies. The aim of the current study was to identify risk factors affecting the QoL of patients with carotid stenosis in stroke prevention.

**Methods:**

Self-sufficient patients (50–80 years of age) with ≥20% carotid artery stenosis followed in the neurosonology laboratory, and without any severe illnesses within the last 12 months, dementia, or psychiatric disorders were selected for the study after signing informed consent. Patients completed two standardized QoL questionnaires (WHOQoL-BREF and EQ-5D-3 L) and a visual pain scale, provided covariate variables (medication, age, gender, education, and social situation), and the blood pressure and body mass indexes were recorded. Logistic regression (forward stepwise method) was used to identify factors affecting the individual domains of QoL questionnaires.

**Results:**

Of the 584 consecutive patients, 502 met the inclusion criteria and 344 completely filled both QoL questionnaires (164 men; mean age, 69.7 ± 7.8 years). An independent predictor of worse QoL in all domains was pain. Independent factors decreasing the QoL were lower level of education and blood pressure in the physical health domain, female gender in the psychological domain, and male gender in the social relationships domain. Independent factors decreasing satisfaction with health status were female gender and higher blood pressure. Factors negatively influencing the satisfaction with the QoL were living alone, lower level of education, and higher diastolic blood pressure (WHOQoL-BREF). Factors negatively influencing mobility were age, male gender, living alone, lower level of education, and higher body mass index (EQ-5D-3 L; *p* < 0.05 in all cases).

**Conclusions:**

Pain, blood pressure, body mass index, education, living alone, gender, and age were associated with the QoL in patients with carotid stenosis.

**Trial registration:**

ClinicalTrials.gov, NCT02360137. Registered on 26 January 2015.

## Background

Atherosclerotic disease is the leading cause of death and morbidity in developed countries in the past decades [1]. The carotid bifurcation and internal *carotid arteries* are sites with a very high *predilection* for the formation of atherosclerotic plaques [2]. Atherosclerotic carotid stenosis is a main cause of stroke [3] and, stroke is the second most common cause of death and the leading cause of disability worldwide [4, 5]. In fact, about 20% of 15 million stroke patients worldwide are in need of medical care and rehabilitation procedures each year after suffering of stroke, and approximately 5.7 million patients die [6–8].

New treatment methods (i.e., intravenous thrombolysis, endovascular treatment, and neurointensive care) have led to a decrease in the number of stroke patients with permanent disability [9–12]. Nevertheless, only about 50% of patients reach full independency after stroke despite of new treatment use [10–12]. The persisting impairment in motor function is the main, but not the only, reason for dependency in activities of daily living among stroke patients [13, 14]. Post-stroke depression, cognitive impairment, urinary incontinence, and other non-motor function impairment are relatively frequent health problems after stroke, thus leading to a decrease in the quality of life (QoL) [13–15]. Thus, QoL has become one of the main endpoints in stroke prevention or acute stroke treatment studies and, evaluation of QoL has become the standard tool for evaluation of the effectiveness of prevention and acute treatment of stroke [16–18].

The prevalence of carotid stenosis is approximately 10% in subjects > 70 years of age, the majority of whom are asymptomatic [19]; however, there are a lack of studies evaluating QoL in patients with carotid stenosis. Moreover, the majority of published studies have only included patients with carotid stenosis indicated for carotid revascularization, e.g., carotid endarterectomy or stenting [20–24]. A systematic review and meta-analysis of studies evaluating QoL after carotid revascularization showed that QoL did not change significantly in any domain in patients 1 year after carotid endarterectomy or stenting. Nevertheless, physical function, vitality, body pain, and social function domains were transiently worse 2 weeks after the procedure, and occurred more frequently after carotid endarterectomy than after carotid stenting [24]. Middleton et al. [25] showed that QoL of patients 3 months after carotid revascularization was better than QoL in the general population of patients with a previous history of stroke, but remained worse than in patients without a previous stroke.

Thus, one may hypothesize that risk factors and clinical consequences of atherosclerosis in patients with carotid stenosis may significantly influence the QoL. Identification of the factors influencing the QoL in a prevention of stroke is necessary for treatment optimization and to preserve QoL. The aim of the current study was to identify risk factors affecting the QoL of patients with carotid atherosclerotic stenosis in stroke prevention.

## Methods

### Questionnaires

A quantitative cross-sectional study with standardized QoL questionnaires (World Health Organization Quality of Life [short version] {WHOQoL-BREF} and three-Level EuroQol-5D [EQ-5D-3 L]) was conducted to identify the factors influencing QoL in patients with carotid atherosclerotic stenosis in stroke prevention including risk factors for atherosclerosis (age, gender, weight, height and body mass index, systolic and diastolic blood pressure, arterial hypertension, diabetes mellitus, hyperlipidemia, smoking and alcohol misuse), diseases caused by atherosclerosis (coronary heart disease, myocardial disease, atrial fibrillation and other heart disease, transient ischemic attack, stroke, and peripheral arterial disease), arterial interventions (carotid endarterectomy, coronary artery bypass graft, surgery for peripheral arterial disease, carotid artery stenting, coronary artery stenting, and stenting of other arteries), and other concomitant factors (pain, social situation, and education). For this purpose, one generic questionnaire (WHOQoL-BREF) and one generic questionnaire widely used in stroke patients (EQ-5D-3 L) were selected [26, 27]. The reason for using two different generic questionnaires was to compare the usability of both questionnaires for identifying risk factors influencing QoL.

The WHOQoL-BREF questionnaire included two questions assessing the individual’s overall perception of QoL and the overall perception of their health, and 24 questions in four domains (physical health – DOM1, psychological – DOM2, social relationships – DOM3, and environment – DOM4). Particular items were assessed using a five-point Likert scale [26]. The mean score of items within each domain was used to calculate the domain score. The mean score of the first two items (How would you rate your quality of life? – Q1, How satisfied are you with your health? – Q2) was calculated separately as defined in WHOQoL User Manual [28]. The official Czech version of the WHOQoL-BREF questionnaire was used with permission from The World Health Organization.

The second questionnaire was the generic questionnaire EQ-5D-3 L [27]. The reason for using this second generic questionnaire was that the second questionnaire has been frequently used in stroke patients and contains different domains in comparison with WHOQoL-BREF. The EQ-5D-3 L contains five domains (questions) involving QoL (mobility – DOM1, self-care – DOM2, usual activities – DOM3, pain/discomfort – DOM4, and anxiety/depression – DOM5). The respondents used a three-level evaluation of the health state description (no problems, some or moderate problems, and an inability to do/extreme problems). The second part of the questionnaire was the visual analogue 100-point scale, which evaluated the current health status of the individual [29]. The official Czech version of the EQ-5D-3 L questionnaire was used with permission from The EuroQol Research Foundation. The three-level EQ-5D questionnaire, instead of the five-level questionnaire, was used due to the non-existence of an official Czech version of EQ-5D-5 L when the study was designed.

### Participants

Participants from the observational stroke prevention study (ANTIQUE Trial, ClinicalTrials.gov Identifier: NCT02360137, registered on January 26, 2015) who were followed in the Neurosonology Laboratory were selected for participation in the study. The inclusion criteria were as follows: a) self-sufficiency with 0–2 points on the modified Rankin scale (mRS); b) carotid atherosclerotic stenosis ≥20% using ECST study criteria [30]; c) 50–80 years of age; d) and signed informed consent. The exclusion criteria were as follows: a) hospitalization for a severe illness, including stroke, during the last 12 months; b) dementia (Mini Mental State Examination < 20 points; c) psychiatric disease, including depression (Beck depression Inventory ≥20 points); d) severe visual or hearing impairment or other inability to complete the questionnaires based on the patient’s judgement; e) terminal stage of the disease including active cancer with a life expectancy < 2 years (according to the physician opinion); and f) living in a retirement home, nursing home, or hospital.

The entire study was conducted in accordance with the Helsinki Declaration of 1975, as revised in 2004 and 2008. The study was approved by the local Ethics Committee of the Faculty of Health Sciences, Palacký University Olomouc (No. UPOL-7279/1040–2015). All subjects provided written informed consent before enrollment.

### Clinical examination

The neurologic and physical examinations, and duplex sonography of the cervical arteries were performed in all patients. The covariate variables (diseases, surgical procedures, medication, age, gender, level of education [primary, secondary, secondary with graduation, and tertiary], social situation [marital status, living alone, living with a partner or with family members], blood pressure, ten-level visual analogue pain scale, body mass index [BMI], sufficiency using mRS, smoking, alcohol consumption [the usual daily dose of alcohol reported by the patient], and percent of carotid stenosis) were recorded. Data were collected from medical and self-reports of patients.

### Statistics

Pre-study calculations (expected difference of 0.5 point in WHOQoL domain for the variable presented in 50% of subjects) showed that a minimum of 502 respondents were required to reach significant results for with an alpha value of 0.05 (two-tailed) and a beta value of 0.8, assuming that 60% of subjects (301 respondents) will pass inclusion criteria and return completely filled questionnaires. Both questionnaires were evaluated as complete when ≤20% of items were missing. Covariate missing value did not exclude the patients from analysis, with the exception of logistic regression.

The normality of data distribution was checked using the Shapiro–Wilk test. All data except body height were not normally distributed. Demographic data are reported as the median, mean and standard deviation or number and percentage. Data from both questionnaires were processed as ordinal data with 5 (WHOQoL-BREF) or 3 (EQ-5D-3 L) values, except for the visual analogue scale in EQ-5D-3 L, in which data were processed as quantitative. Categorical variables in the two arms (completers and non-completers) were compared by Fisher’s exact test. Continuous variables were compared by the Student’s *t-*test for normally distributed values. The Mann–Whitney *U* test (for variables with 2 groups) or Kruskal-Wallis test (for variables with more than 2 groups) was used. The Spearman correlation coefficient was calculated for evaluation of the correlation between factors with qualitative or ordinal quantities and questions or domains of QoL questionnaires. Logistic regression (forward stepwise method) was used to identify factors affecting the individual domains of QoL questionnaires (separate multivariable logistic model for each domain or question; totally 12 models). The following variables were used for logistic regression analysis: age (quantitative data); gender (qualitative data); marital status (semi-quantitative data); social situation (semi-quantitative data); level of education (semi-quantitative data); presence of arterial hypertension, diabetes mellitus, hyperlipidemia, coronary heart disease, or atrial fibrillation; history of myocardial infarction, other heart disease, stroke, transient ischemic attack, carotid endarterectomy, carotid artery stenting, coronary artery bypass graft, surgery for peripheral arterial disease, coronary artery stenting (all qualitative data; combination of self-reports and medical reports); smoking (self-report); alcohol consumption (self-report; 1 international unit = 10 mL of pure alcohol); BMI; systolic blood pressure; diastolic blood pressure; visual pain scale (all quantitative data). The quantitative values of the 4 domains in WHOQoL-BREF were dichotomized with a cut-off value of 13, Q1 and Q2 in WHOQoL-BREF with a cut-off value of 3 (1 + 2 vs. 3 + 4 + 5), 5 domains in EQ-5D-3 L with a cut-off value of 2 (1 vs. 2 + 3), and the visual analogue scale in EQ-5D-3 L with a cut-off value of 51.

All tests were carried out at an alpha level of significance of 0.05. All data were analyzed using IBM SPSS Statistics (v22.0; SPSS, Inc., Chicago, IL, USA).

## Results

**Fig. 1:**
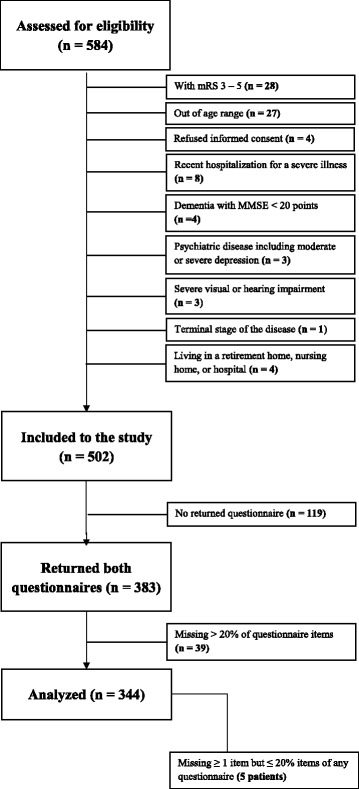
Study flow chart. mRS – modified Rankin score; MMSE – Mini-Mental State Examination

Of the 584 consecutive patients examined in the Neurosonology Laboratory, 502 met the inclusion criteria, and 344 completed both QoL questionnaires (164 men; mean age, 69.7 ± 7.8 years) over a 3-month interval (April–June 2016) – Fig. 1. Demographic data are presented in Table 1. There was no statistically significant difference in any demographic parameter between completers (patients who completed the questionnaires) and non-completers (*p* < 0.05 for all items). Cronbach’s alpha for particular subscales in WHOQoL-BREF in the presented study varied between 0.73 and 0.82. Cronbach’s alpha for EQ-5D-3 L was 0.74.

**Table 1:** Demographic data of patients selected for the study, completers (patients who completed the questionnaires) and non-completers (patients excluded from the study)

		Patients selected for the study (*n* = 502)	Completers (344 patients)	Non-completers (*n* = 158)	*P* value*
Male gender; n (%)		260 (51.8)	164 (47.7)	96 (60.8)	0.006^a^
Age, years; median, mean ± SD		71, 70.0 ± 7.6	71, 69.5 ± 7.8	72, 70.8 ± 7.0	0.069^b^
Weight, kg; median, mean ± SD		81, 81.1 ± 15.3	80, 80.4 ± 14.9	82, 82.6 ± 15.9	0.144^b^
Height, cm; median, mean ± SD		168, 168.9 ± 8.4	169, 169.0 ± 8.3	169, 169.2 ± 8.6	0.523^b^
Body mass index; median, mean ± SD		28, 28.3 ± 4.4	28, 28.1 ± 4.2	29, 28.7 ± 4.7	0.357^b^
Systolic blood pressure, mm Hg; median, mean ± SD		135, 135.1 ± 12.4	135, 134.8 ± 12.3	136, 135.6 ± 12.6	0.412^b^
Diastolic blood pressure, mm Hg; median, mean ± SD		80, 79.4 ± 8.3	80, 79.3 ± 8.4	80, 79.6 ± 8.2	0.498^b^
Visual pain scale; median, mean ± SD		4, 3.8 ± 2.5	4, 3.9 ± 2.5	4, 3.6 ± 2.5	0.168^b^
Arterial hypertension; n (%)		429 (85.5)	141 (89.2)	141 (89.2)	0.083^a^
Diabetes mellitus; n (%)		115 (22.9)	39 (24.7)	39 (24.7)	0.528^a^
Hyperlipidemia; n (%)		268 (53.4)	191 (55.5)	77 (48.7)	0.294^a^
Coronary heart disease; n (%)		156 (31.1)	99 (28.8)	57 (36.1)	0.109^a^
Myocardial infarction; n (%)		49 (9.8)	35 (10.2)	14 (8.9)	0.639^a^
Atrial fibrillation; n (%)		66 (13.1)	43 (12.5)	23 (14.6)	0.711^a^
Other heart disease; n (%)		53 (10.6)	32 (9.3)	21 (13.3)	0.203^a^
Transient ischemic attack; n (%)		57 (11.4)	42 (12.2)	15 (9.5)	0.624^a^
Stroke; n (%)		217 (43.2)	145 (42.2)	72 (45.6)	0.535^a^
Surgery/stenting of arteries; n (%)		106 (21.1)	79 (23.0)	27 (17.1)	0.120^a^
Smoking; n (%)		62 (12.4)	49 (14.2)	13 (8.2)	0.057^a^
Social situation; n (%)	Living alone	111 (22.1)	72 (21.0)	39 (24.7)	0.268^c^
	Living with partner	94 (18.7)	60 (17.4)	34 (21.5)	
	Living with family	297 (59.2)	212 (61.6)	85 (53.8)	
Education; n (%)	Primary	106 (21.1)	67 (19.5)	39 (24.7)	0.152^c^
	Secondary without graduation	146 (29.1)	104 (30.2)	42 (26.6)	
	Secondary with graduation	160 (31.9)	115 (33.4)	45 (28.5)	
	Tertiary	90 (17.9)	58 (16.9)	32 (20.3)	
Alcohol consumption, units/day; n (%)	0	253 (50.4)	170 (49.4)	83 (52.5)	0.244^c^
	1	143 (28.5)	109 (31.7)	34 (21.5)	
	2	100 (19.9)	60 (17.4)	40 (25.3)	
	≥ 3	6 (1.2)	5 (1.5)	1 (0.6)	

n – number; SD – standard deviation; * global test comparing the distributions of completers and non-completers; ^a^ Fisher’s exact test; ^b^ Student’s *t-*test; ^c^– Kruskal-Wallis test

The correlations between observed factors and QoL in particular domains are shown in Table 2. Factors negatively influencing the QoL were identified using the forward stepwise method of multiple logistic regression and are presented in Tables 3 and 4.

**Table 2:** Correlation between questions or domains of quality of life questionnaires and evaluated risk factors

	WHO-QOL BREF						EQ-5D-3 L					
	Q1	Q2	DOM1	DOM2	DOM3	DOM4	DOM1	DOM2	DOM3	DOM4	DOM5	Health status
Age^a^	−0.011	−0.013	− 0.124*	−0.088	− 0.066	0.099	0.127*	0.101	0.118*	0.086	0.022	−0.081
Gender^b^	0.249	0.074	0.938	0.607	0.020	0.974	0.050	0.064	0.043	0.301	0.015	0.055
Spouse^b^	0.020	0.070	0.090	0.777	0.350	0.340	0.031	0.417	0.836	0.026	0.304	0.651
Living alone^c^	0.002	0.108	0.014	0.507	0.154	0.045	0.066	0.799	0.262	0.014	0.040	0.344
Education^a^	0.266*	0.104	0.255*	0.195*	0.129*	0.227*	−0.190*	−0.096	− 0.143*	− 0.180*	− 0.080	0.172*
Arterial hypertension^b^	0.639	0.889	0.376	0.768	0.321	0.536	0.060	0.336	0.061	0.465	0.457	0.429
Diabetes mellitus^b^	0.642	0.052	0.106	0.912	0.108	0.351	0.102	0.052	0.253	0.098	0.228	0.006
Hyperlipidemia^b^	0.691	0.463	0.053	0.222	0.851	0.434	0.057	0.616	0.108	0.472	0.927	0.464
Coronary heart disease^b^	0.411	0.529	0.087	0.925	0.507	0.204	0.108	0.514	0.618	0.054	0.727	0.228
Atrial fibrillation^b^	0.186	0.423	0.837	0.146	0.986	0.137	0.915	0.733	0.935	0.778	0.323	0.852
Myocardial infarction^b^	0.529	0.778	0.551	0.574	0.591	0.116	0.622	0.303	0.142	0.492	0.105	0.544
Other heart disease^b^	0.315	0.611	0.877	0.192	0.154	0.059	0.954	0.701	0.890	0.571	0.521	0.727
Stroke/TIA^b^	0.102	0.726	0.166	0.444	0.212	0.664	0.452	0.810	0.379	0.529	0.120	0.315
Carotid endarterectomyb	0.055	0.782	0.946	0.343	0.095	0.403	0.253	0.269	0.704	0.415	0.318	0.661
CABG^b^	0.649	0.218	0.729	0.248	0.832	0.752	0.073	0.516	0.804	0.454	0.248	0.106
Surgery for PAD^b^	0.051	0.441	0.173	0.229	0.778	0.301	0.337	0.435	0.505	0.309	0.051	0.204
Carotid artery stenting^b^	0.793	0.660	0.425	0.860	0.780	0.766	0.580	0.109	0.119	0.381	0.444	0.748
Coronary artery stenting^b^	0.623	0.869	0.611	0.980	0.750	0.224	0.183	0.825	0.430	0.903	0.960	0.256
Smoking^b^	0.581	0.799	0.824	0.595	0.539	0.265	0.074	0.401	0.699	0.827	0.197	0.463
Alcohol abuse^b^	0.555	0.164	0.099	0.581	0.829	0.256	0.268	0.133	0.213	0.115	0.615	0.700
Body mass index^a^	0.023	−0.036	−0.060	0.039	0.065	0.014	0.137*	−0.061	0.025	0.050	0.038	−0.027
Systolic blood pressure^a^	−0.117*	−0.148*	−0.138*	−0.154*	−0.112*	−0.109*	0.108*	0.110*	0.108*	0.085	0.096	−0.174*
Diastolic blood pressure^a^	−0.125*	− 0.192*	−0.150*	− 0.159*	−0.129*	− 0.112*	0.112*	0.130*	0.102	0.065	0.065	−0.172*
Visual pain scale^a^	−0.306*	−0.297*	− 0.637*	−0.432*	− 0.328*	−0.377*	0.445*	0.214*	0.338*	0.556*	0.360*	−0.441*

Used method, value: ^a^ – Spearmann correlation, Spearman correlation coefficient (r); ^b^ – Mann-Whitney U-test, *P* value; ^c^ – Kruskal-Wallis test, *P* value; WHO-QOL BREF – World Health Organization Quality of Life short version; EQ-5D-3 L – the 3-item EuroQol-5D; TIA – transient ischemic attack; CABG – coronary artery bypass graft; PAD – peripheral artery disease; WHO-QOL: Q1 – overall perception of quality of life; Q2 – the overall perception of their health; DOM1 – physical health domain; DOM2 – psychological domain; DOM3 – social relationships domain; DOM4– environment domain; EQ-5D-3 L: DOM1 – mobility domain; DOM2 – self-care domain; DOM3– usual activities domain; DOM4 – pain/discomfort domain; DOM5– anxiety/depression domain; * - *P* < 0.05

**Table 3:** Factors affecting the individual domains of quality of life in WHOQoL-BREF questionnaire – logistic regression, forward stepwise method, separate model for each question or domain

Question/ Domain	Factor^a^	OR	95 CI	*P* value
Q1	Social situation			
	- living alone	reference		
	- living with a family member	1.649	0.734–3.705	0.226
	- living with a partner	2.509	1.349–4.972	0.004
	Education level			
	- primary	*reference*		
	- secondary without graduation	1.177	0.578–2.397	0.653
	- secondary with graduation	2.572	1.206–5.484	0.014
	- tertiary	4.351	1.461–12.957	0.008
	Diastolic blood pressure (per 10 mmHg)	0.672	0.477–0.956	0.023
	Pain (per 1 point in the Visual pain scale)	0.852	0.759–0.956	0.007
Q2	Gender			
	- female	*reference*		
	- male	1.784	1.117–2.850	0.015
	Diastolic blood pressure (per 10 mmHg)	0.477	0.341–0.667	< 0.001
	Pain (per 1 point in the Visual pain scale)	0.798	0.724–0.879	< 0.001
DOM 1	Education level			
	- primary	*reference*		
	- secondary without graduation	2.472	1.125–5.432	0.024
	- secondary with graduation	2.956	1.364–6.406	0.006
	- tertiary	1.871	0.749–4.675	0.180
	Diastolic blood pressure (per 10 mmHg)	0.961	0.928–0.995	0.027
	Pain (per 1 point in the Visual pain scale)	0.593	0.519–0.678	< 0.001
DOM 2	Gender			
	- female	*reference*		
	- male	1.910	1.016–3.591	0.044
	Pain (per 1 point in the Visual pain scale)	0.673	0.585–0.773	< 0.001
DOM 3	Gender			
	- female	*reference*		
	- male	0.543	0.297–0.994	0.048
	Pain (per 1 point in the Visual pain scale)	0.785	0.692–0.890	< 0.001
DOM 4	Pain (per 1 point in the Visual pain scale)	0.619	0.511–0.750	< 0.001

^a^- only factors significantly influencing the corresponding question or domain with *p* < 0.05 are mentioned; WHO-QOL BREF – World Health Organization Quality of Life short version; EQ-5D-3 L – the 3-item EuroQol-5D; WHO-QOL: Q1 – overall perception of quality of life; Q2 – the overall perception of their health; DOM1 – physical health domain; DOM2 – psychological domain; DOM3 – social relationships domain; DOM4 – environment domain

**Table 4:** Factors affecting the individual domains of quality of life in EQ-5D-3 L questionnaire – logistic regression, forward stepwise method

Question/ Domain	Factor^a^	OR	95 CI	*P* value
DOM1	Age (per 1 year)	0.962	0.929–0.996	0.028
	Gender			
	- female	*reference*		
	- male	0.492	0.285–0.851	0.011
	Social situation			
	- living alone	reference		
	- living with a family member	1.322	0.775–2.758	0.152
	- living with a partner	2.037	1.052–3.953	0.035
	Education level			
	- primary	*reference*		
	- secondary without graduation	1.201	0.610–2.219	0.385
	- secondary with graduation	1.448	1.108–1.893	0.007
	- tertiary	1.319	0.698–2.441	0.208
	Body mass index (per 1 unit)	0.895	0.839–0.955	0.001
	Pain (per 1 point in the Visual pain scale)	0.638	0.567–0.718	< 0.001
DOM2	Pain (per 1 point in the Visual pain scale)	0.750	0.646–0.871	< 0.001
DOM3	Pain (per 1 point in the Visual pain scale)	0.718	0.644–0.801	< 0.001
DOM4	Pain (per 1 point in the Visual pain scale)	0.505	0.429–0.594	< 0.001
DOM5	Gender			
	- female	*reference*		
	- male	1.741	1.089–2.783	0.021
	Pain (per 1 point in the Visual pain scale)	0.721	0.651–0.798	< 0.001
Health status	Education level			
	- primary	*reference*		
	- secondary without graduation	1.098	0.504–1.944	0.612
	- secondary with graduation	1.332	1.041–1.705	0.023
	- tertiary	1.297	0.649–2.168	0.428
	Systolic blood pressure (per 10 mmHg)	0.787	0.650–0.953	0.014
	Pain (per 1 point in the Visual pain scale)	0.782	0.708–0.864	< 0.001

^a^- only factors significantly influencing the corresponding question or domain with *p* < 0.05 are mentioned; EQ-5D-3 L: DOM1 – mobility domain; DOM2 – self-care domain; DOM3 – usual activities domain; DOM4 – pain/discomfort domain; DOM5 – anxiety/depression domain

In the WHOQoL-BREF questionnaire, pain was identified as an independent predictor of worse QoL in all domains and questions (OR per 1 unit in the visual pain scale = 0.593–0.852, *p* < 0.01 for all cases) – Table 3. Furthermore, the factors influencing the overall perception of QoL (Q1) were living with a partner (OR = 2.509, *p* = 0.004), level of education (OR = 2.572 for secondary with graduation, *p* = 0.014, OR = 4.351 for tertiary, *p* = 0.008), and diastolic blood pressure (OR = 0.672, *p* = 0.023). Male gender was identified as an independent factor positively influencing the overall perception of health (Q2; OR = 1.784, *p* = 0.015) and, the psychological domain (DOM2; OR = 0.910, *p* = 0.044), and negatively influencing the social relationships domain (DOM 3; OR = 0.543, *p* = 0.048). Diastolic blood pressure independently influenced the QoL in the overall perception of health (Q2; OR = 0.477, *p* < 0.001) and the physical health domain (DOM1; OR = 0.961, *p* = 0.027).

In the EQ-5D-3 L questionnaire, the independent predictor of worse QoL in all domains and current health status was pain (OR per 1 level in the 10-level visual analogue pain scale = 0.505–0.787, *p* < 0.01 for all cases) – Table 4. Moreover, age (OR = 0.962, *p* = 0.028), gender (OR = 0.492 for male gender, *p* = 0.011), living with a partner (OR = 2.037, *p* = 0.035), education level (OR = 1.448 for secondary with graduation, *p* = 0.007), and BMI (OR = 0.895, *p* = 0.001) were identified as factors independently influencing the mobility domain (DOM1). Male gender (OR = 1.741, *p* = 0.021) positively influenced the anxiety/depression domain (DOM 5). The education level (OR = 1.332 for secondary with graduation, *p* = 0.023) and systolic blood pressure (OR = 0.787, *p* = 0.014) were identified as independent factors influencing the current health status measured on the visual analogue scale.

A history of stroke, transient ischemic attack, myocardial infarction, arterial hypertension, diabetes mellitus, hyperlipidemia, coronary heart disease, atrial fibrillation, arterial surgery, stenting, smoking, and alcohol consumption had no significant influence on QoL in both questionnaires (*p* > 0.05 for all cases).

## Discussion

The present study demonstrated that a history of vascular events (stroke, transient ischemic attack, coronary heart disease, and myocardial infarction), risk factors influencing progression of atherosclerosis (arterial hypertension, diabetes mellitus, hyperlipidemia, smoking, and alcohol consumption), and vascular interventions for atherosclerotic stenoses were not associated with QoL in self-sufficient patients with carotid atherosclerotic stenosis and without dementia or moderate or severe depression. The only factors influencing the QoL in these patients were pain, blood pressure, and BMI, living situation, level of education, age and gender. Thus, the current patient’s situation and health status, but not the medical history were the main factors influencing the evaluation of QoL in these patients.

The interesting result of our study was that the presence of arterial hypertension was not identified as a factor influencing the QoL in both questionnaires, in contrast to actual blood pressure, which was negatively correlated with satisfaction with health status, satisfaction with the QoL, and physical health domain evaluation measured on the WHOQoL-BREF, and the current health status measured on the EQ-5D-3 L. Lower blood pressure was associated with a better QoL and a better sense of patient well-being, as in previous studies [31, 32]. Obesity represents another factor with potential influence on the QoL [33, 34]. BMI was identified as a factor negatively correlated with QoL in the mobility domain measured on the EQ-5D-3 L in our study. Ford et al. [35] showed, also, that increased BMI significantly impaired health-related QoL and affected a physical functioning more strongly than mental functioning.

Social situation was a factor influencing the overall perception of QoL measured on the WHOQoL-BREF and mobility measured on the EQ-5D-3 L. Patients living alone scored significantly worse in both domains. Loneliness is a known factor negatively influencing QoL in chronically ill patients or stroke survivors [36–38].

In agreement with other studies, pain was identified as a strong independent predictor of lower QoL in all domains of both questionnaires in our study [39–41].

Gender was identified as a factor significantly influencing QoL in the psychological domain and satisfaction with present health status (worse in females), and social relationships domains (worse in males) measured on the WHOQoL-BREF, and mobility measured on the EQ-5D-3 L. The results of published studies evaluating the influence of gender on QoL are inconclusive. Jönsson et al. [42] reported that female gender is associated with higher scores for the physical role, emotional function, and the general health in stroke survivors. In contrast, van Eeden et al. [43] demonstrated higher QoL in males compared to females 2, 6, and 12 months after stroke; however, it should be pointed out that not only post-stroke patients were enrolled in our study.

Age was the second non-modifiable factor influencing the QoL. Nevertheless, age only correlated significantly with QoL in the mobility domain measured on the EQ-5D-3 L. A recently published Dutch study confirmed that age influenced the elderly predominantly in the mobility domain of all domains in the EQ-5D-3 L questionnaire [44].

The last identified factor influencing the QoL was level of education. Level of education was negatively correlated with the overall perception of QoL measured on the WHOQoL-BREF, satisfaction of present health status, and QoL in the mobility domain measured on the EQ-5D-3 L. The World Health Organization has determined education to be one of the social determinants of health because low education levels are linked with poor health, more stress and lower self-confidence [45]. Education has also been identified as an independent factor positively influencing QoL in the study performed by Vlajinac et al. [22].

The severity and character of persisting neurologic deficits after stroke could be additional factors influencing the QoL in patients with carotid stenosis [46–49]. A Korean study showed that patients with stroke and facial palsy evaluated their QoL worse than patients with dysarthria [47]. Also, persistent visual deficits, hemiparesis, and recurrent stroke could influence the QoL significantly [48, 49]. We did not identify persistent neurologic deficits as a factor influencing QoL in patients after stroke if self-sufficient. Nevertheless, the character and severity of neurologic deficits were not evaluated in the present study.

Comparing the ability of both questionnaires to identify factors influencing QoL, the EQ-5D-3 L questionnaire identified not only the same five independent factors (gender, level of education, living alone, pain, and blood pressure) as the WHOQoL-BREF questionnaire, but two additional factors (age and body mass index). Furthermore, the EQ-5D-3 L questionnaire consisted of only 5 questions and 1 visual analogue scale in comparison with 26 questions in the WHOQoL-BREF. These results showed that the EQ-5D-3 L questionnaire is more suitable than the WHOQoL-BREF for patients with carotid stenosis.

The main limitation of the study was patient selection. We enrolled only self-sufficient patients visiting the Neurosonology Laboratory for the evaluation of atherosclerosis of the carotid arteries. Thus, patients with other etiologies of stroke could be neglected. The second limitation was the monocentric character of the study. Third, patients recently hospitalized for a severe illness, patients with dementia, psychiatric disease, including moderate or severe depression, severe visual or hearing impairment, patients in a terminal stage of the disease, and patients living in a retirement home, nursing home, or hospital were excluded to avoid uncontrolled bias. Only 4% of screened patients were excluded due to these reasons. Thus, the results should be generalizable. Nevertheless, in further studies, the extension of inclusion criteria, recorded variables and sample size may enable enrollment of a more heterogenous group of patients with carotid stenosis and may subsequently identify more predictors of QoL.

## Conclusion

Pain, blood pressure, BMI, education, living alone, gender, and age, but not a previous stroke or myocardial infarction, affect the QoL in self-sufficient patients with carotid stenosis without dementia or severe depression. Thus, current social and health status factors should be recorded in studies with carotid stenosis patients. Awareness and understanding of the factors influencing QoL in patients with carotid stenosis should be important to support a maintained or increased QoL and may also lead to more holistic management and patient care.

## Abbreviations

BMI: Body mass index
EQ-5D-3 L: 3-Level EuroQol-5D
QoL: Quality of life
WHOQoL-BREF: World Health Organization Quality of Life [short version]
